# Transcriptomes reveal the involved genes in the sea urchin *Mesocentrotus* *nudus* exposed to high flow velocities

**DOI:** 10.1038/s41598-022-17793-w

**Published:** 2022-08-05

**Authors:** Ruihuan Tian, Dongtao Shi, Donghong Yin, Fangyuan Hu, Jun Ding, Yaqing Chang, Chong Zhao

**Affiliations:** 1 Key Laboratory of Mariculture & Stock Enhancement in North China’s Sea, Ministry of Agriculture and Rural Affairs, Dalian Ocean University, Dalian, 116023 China; 2 Southern Marine Science and Engineering Guangdong Laboratory, Guangzhou, 511458 China

**Keywords:** Gene expression analysis, Sequencing, Marine biology, Biooceanography

## Abstract

Despite the importance of flow velocity in marine ecosystems, molecular mechanisms of the water flow induced behavioral and growth changes remain largely unknown in sea urchins. The present study compared the gene expressions of the sea urchin *Mesocentrotus* *nudus* at high flow velocities (10 cm/s and 20 cm/s) and low flow velocity (2 cm/s) using transcriptomes. A total of 490 and 470 differentially expressed genes (DEGs) were discovered at 10 cm/s and 20 cm/s, respectively. There were 235 up-regulated and 255 down-regulated genes at 10 cm/s, 213 up-regulated and 257 down-regulated genes at 20 cm/s, compared with sea urchins at 2 cm/s. Further, there were 72 overlapped DEGs involved in regulation at both 10 cm/s and 20 cm/s. Gene Ontology (GO) functional annotation showed that DEGs were mainly enriched to cellular process, cell part, binding, and metabolism process. Kyoto Encyclopedia of Genes and Genomes (KEGG) pathway analysis found that DEGs were enriched in three pathways related to amino acid metabolism and lipid metabolism. A number of genes related to growth and metabolism of sea urchins were mobilized in high flow velocity environment. We further highlighted a muscle-associated gene *ankyrin-1*, which is correlated with the movement of tube feet at different flow velocities. The present study provides valuable information on the molecular mechanisms of changed behaviors and growth when sea urchins are exposed to high flow velocity.

## Introduction

The sea urchin *Mesocentrotus* *nudus* naturally distributes in the intertidal and shallow sea of northern China, the Russian Far East, northern Japan, and the Korean Peninsula^1,2^. It is an ecologically and commercially important marine species^3,4^, playing a crucial role in the benthic as well as pelagic food webs^4^. The natural stock of sea urchins is greatly declining with the increasing consumer market, which consequently damages the marine ecosystems^5,6^. Stock enhancement is an effective approach to producing sea urchins, in which small sea urchins (test diameter > 10 mm) are commonly seeded into the sea floor and subsequently harvested at commercial size^7^. Water flow is an important factor affecting the production efficiency of sea urchins, because it is an inevitable abiotic factor in complex marine benthic environments^8–10^.

High flow velocity greatly affects movement and growth of many marine organisms, including fish^11^, shellfish^12^, and echinodermata^13,14^. For example, the growth rate of the turbot *Scophthalmus* *maximus*^15^ and the scallop *Argopecten* *irradians*^16^ showed an upward trend followed by a downward trend when being exposed to the increasing flow velocity. Growth rate^14^ and movement speed^17^ significantly reduced when sea urchins were exposed to a long-term high flow velocity environment. Our previous study found that test diameter and body weight of *M*. *nudus* increased significantly slower at a high flow velocity^18^. Tube feet of sea urchins are highly sensory^19^. Tube feet displayed higher density and better tenacity with the increase of flow velocity^20^. The adhesion ability of sea urchins strengthened at high flow velocity^21,22^, which is strongly related to foraging behavior and risk avoidance of sea urchins^23^. These studies indicate that growth and behaviors of sea urchins probably are malleable when they are exposed to high flow velocity. However, little information is known that the molecular mechanisms of sea urchins exposed to high flow velocity. The transcriptome is an effective technique to understand the changes in gene expressions of organisms in exposure to various environmental stress^24,25^.

Here, sea urchins were exposed to difference flow velocities (2 cm/s, 10 cm/s, and 20 cm/s) for 70 days. The main purpose of the present study is to investigate the effects of long-term flow velocities on gene expressions of *M*. *nudus* using transcriptomes. This provides valuable resources for molecular mechanisms of changed behaviors and growth when sea urchins are exposed to high flow velocity environments.

## Results

### Transcriptome sequencing and assembly

**Figure 1:**
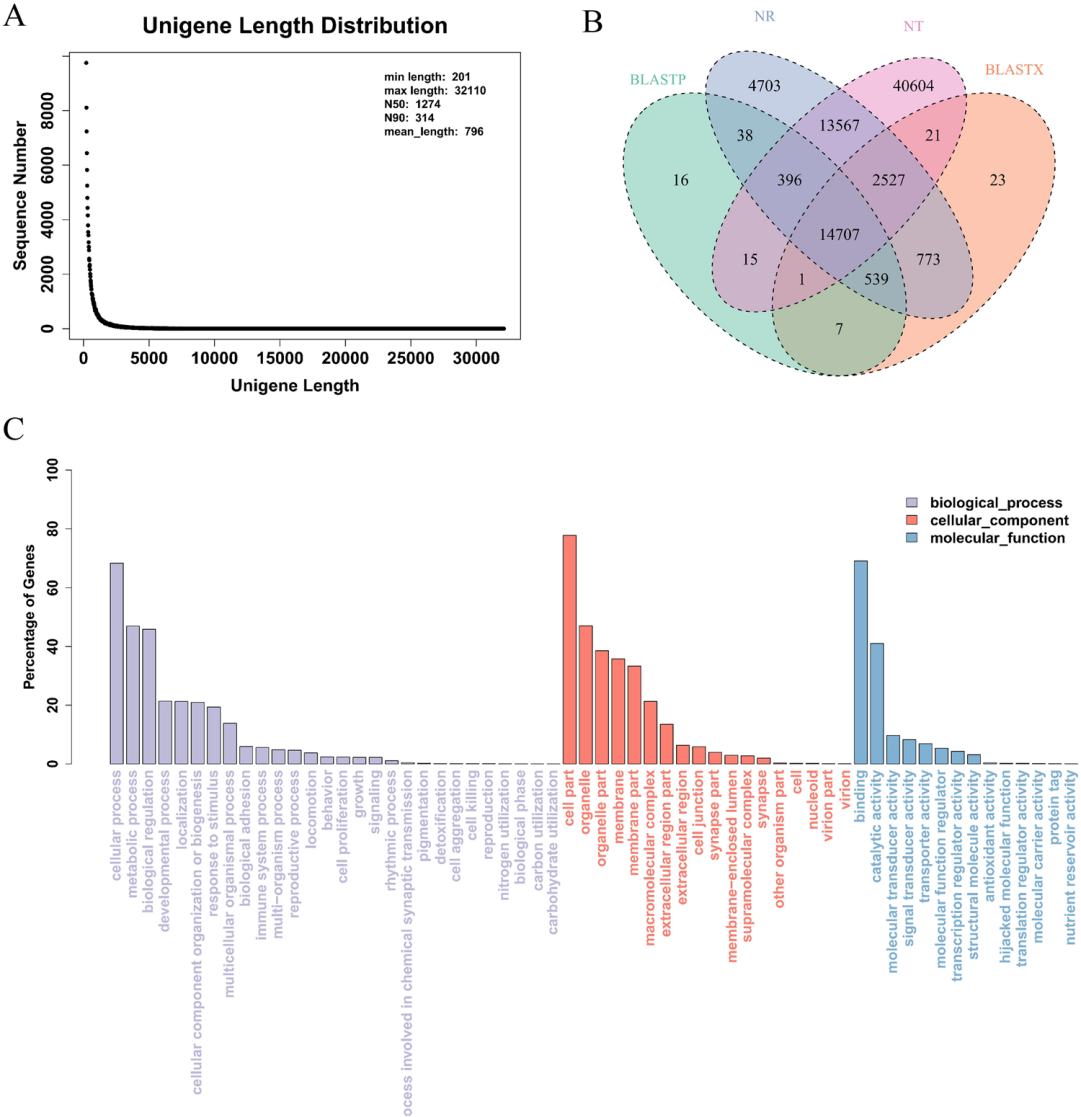
Length distribution of unigenes (**A**). The summary statistics of transcriptome annotation in tube feet of *Mesocentrotus* *nudus* (**B**). Gene Ontology annotation of unigenes in tube feet of *M.* *nudus* (**C**). X-axis represents the terms under biological process, cellular component and molecular function. Y-axis indicates the percentage of unigenes in a specific function cluster.

A total of 432,777,420 raw reads were sequenced from nine samples. 403,298,480 (93.17%) clean reads were assembled into 156,177 unigenes, in which the average length was 796 bp, mainly distributed in 200–1200 bp (Fig. 1A) and GC-content was 38.39%. There were 82.39% of the reads (271,194,264) compared to the transcript sequences. Benchmarking Universal Single-Copy Ortholog (BUSCO) (v3.0.1) evaluation was performed on the assembled unigenes and 94.72% of transcripts were compared with BUSCO groups.

### Gene function annotation and classification

The comparison results of unigenes in various databases are shown in Table 1. There were 37,250, 18,934, and 24,546 unigenes annotated Nr (NCBI non-redundant protein sequences), GO (Gene Ontology), and Swiss-Prot (a manually annotated and reviewed protein sequence database), respectively (Fig. 1B).

**Table 1:** Annotation of unigenes.

	Number of unigenes	Percentage (%)
PFAM	15,588	9.981
TmHMM	5270	3.374
eggNOG	14,882	9.529
RNAMMER	8	0.005
BLASTP	15,719	10.065
BLASTX	18,598	11.908
Map	6291	4.028
Prot	24,546	15.717
NT	71,838	45.998
NR	37,250	23.851
GO	18,934	12.123
KO	10,689	6.844
SignalP	2026	1.297
Total unigenes	156,177	100

The transcriptome assembly was annotated and categorized based on GO (Fig. 1C). In the biological process category, unigenes were mainly annotated in cellular process, metabolic process, and biological regulation. In cell composition, they were mainly concentrated in cell part and organelle. The molecular functions were mainly enriched in binding and catalytic activity.

**Figure 2:**
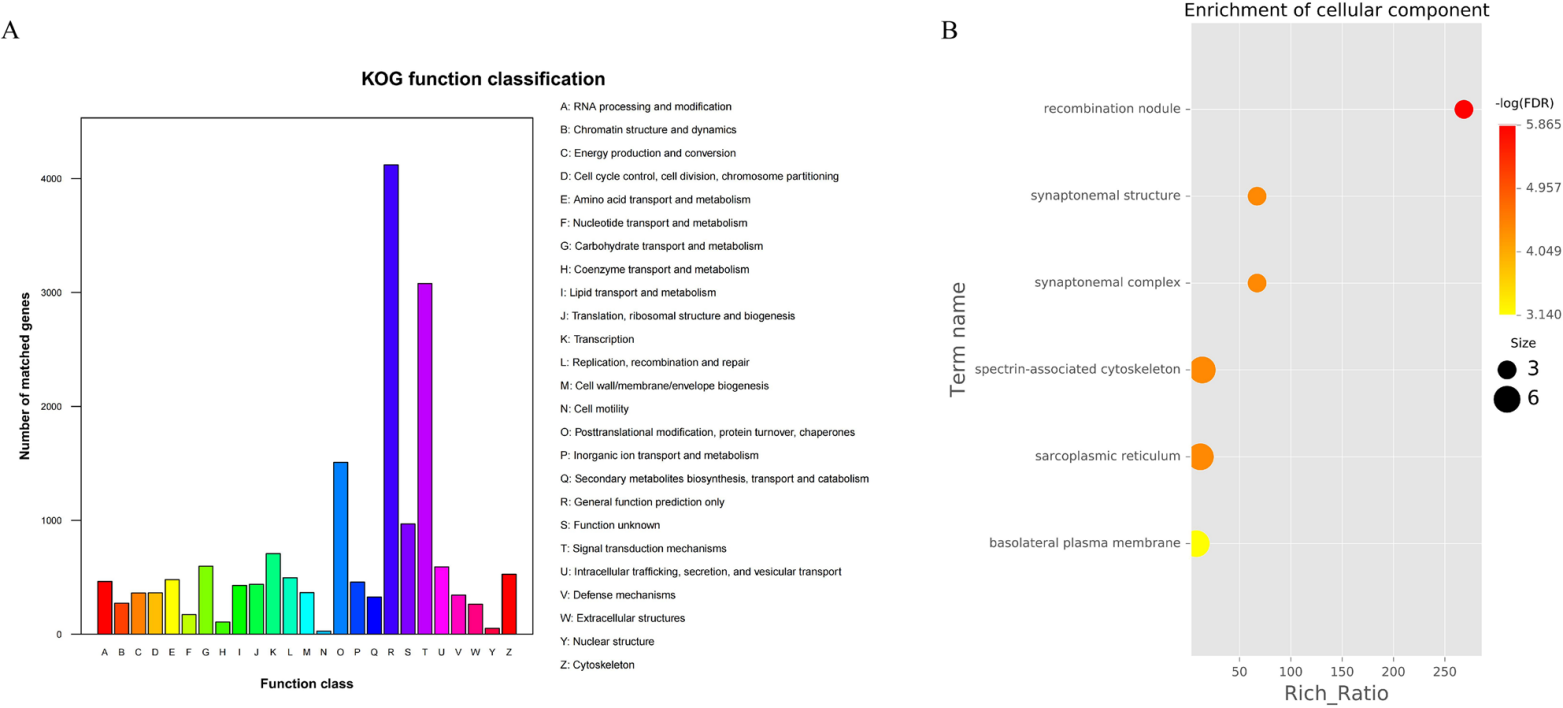
KOG function classification (**A**). The horizontal axis shows the function class, while the vertical axis shows the number of matched unigenes. GO enrichment analysis (cellular component) of DEGs in *Mesocentrotus* *nudus* at 20 cm/s compared to 10 cm/s (**B**).

KOG annotation results are showed in Fig. 2A. Most genes were annotated in general function prediction only, signal transduction mechanisms, and posttranslational modification, protein turnover, associated protein.

### Differential expression analysis

**Figure 3:**
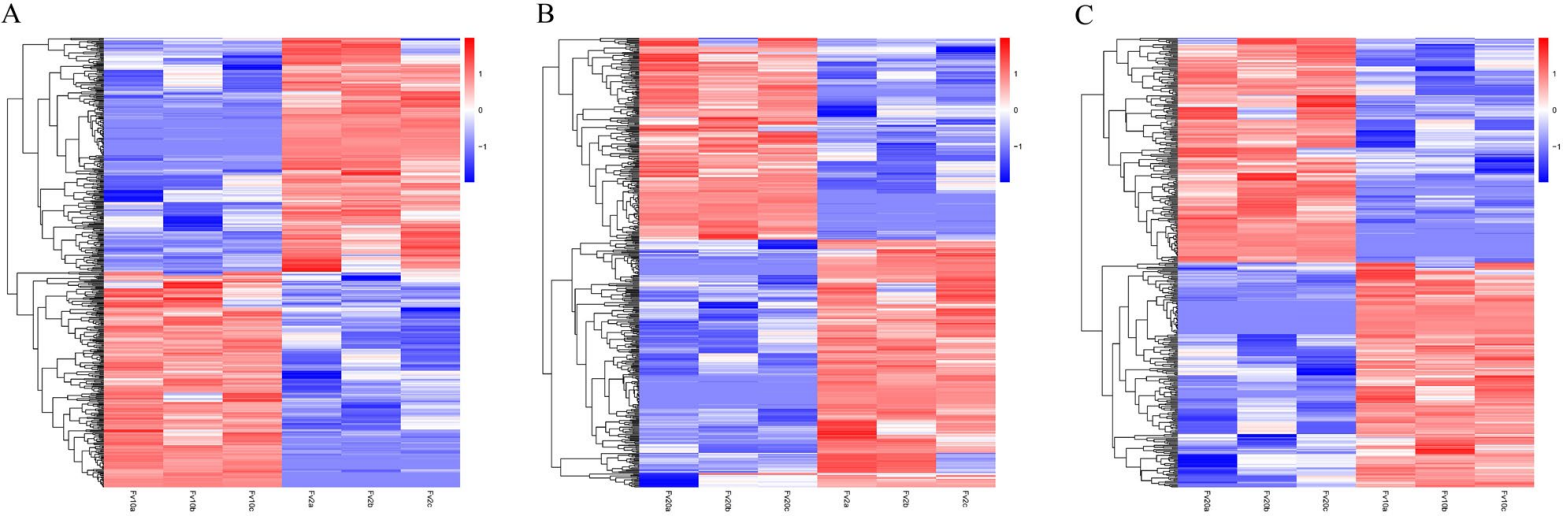
Heat map of the DEGs in transcriptomes of tube feet of *Mesocentrotus* *nudus* at different flow velocities: 10 cm/s vs 2 cm/s (**A**), 20 cm/s vs 2 cm/s (**B**) and 20 cm/s vs 10 cm/s (**C**). Log_2_FPKM is used for clustering. The red represents high-expression gene, the blue represents low-expression gene, and the red to the blue represents high-low expression.

**Figure 4:**
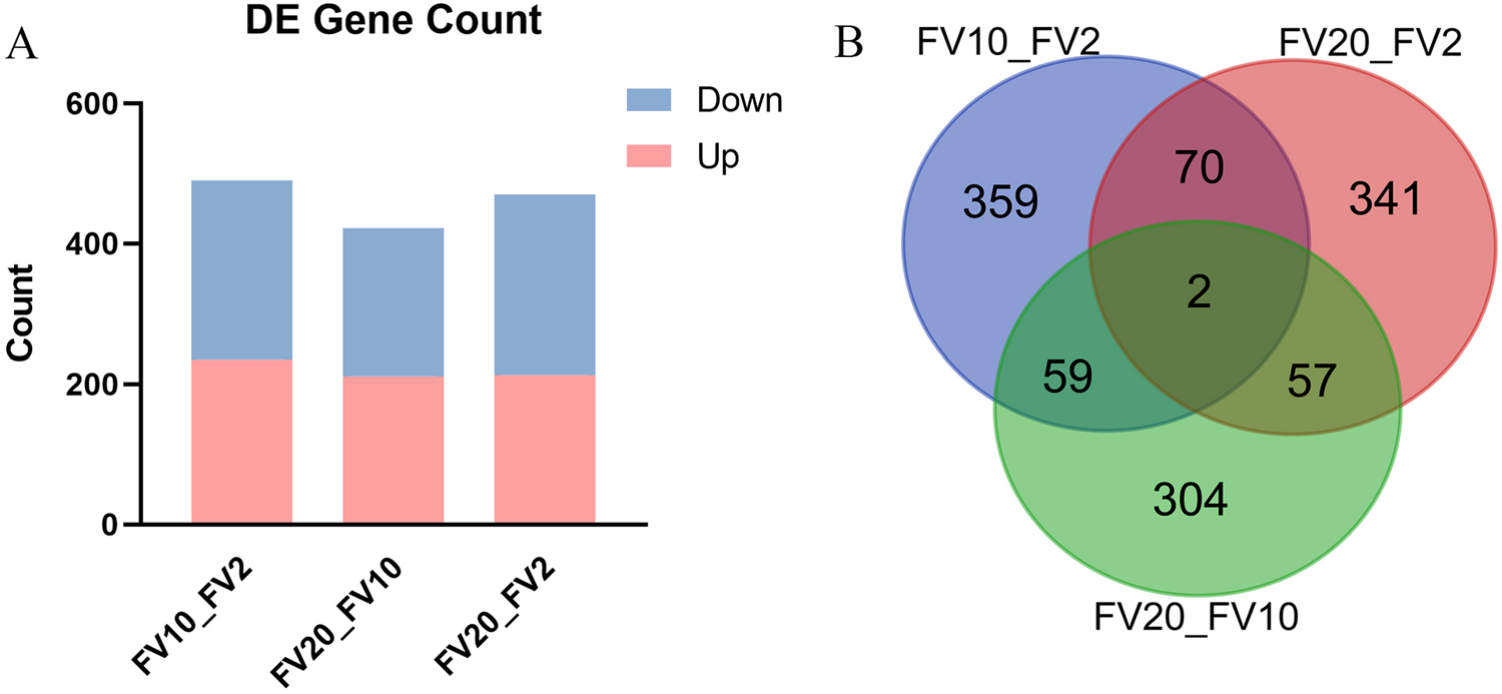
Differentially expressed unigenes analysis of tube feet of *Mesocentrotus* *nudus* at 2 cm/s, 10 cm/s and 20 cm/s (**A**). The horizontal axis represents the control group and vertical axis is the number of DEGs. The DEGs in different transcriptome comparisons (**B**).

RNA-Seq was used to estimate the expression levels of transcripts in each group^26^. DESeq2 (v1.6.3) was used to analyze the differences in gene expression of sea urchins at different flow velocities. The clustering results of DEGs are displayed in Fig. 3. Compared with the sea urchins at 2 cm/s, there were 235 DEGs up-regulated and 255 DEGs down-regulated at 10 cm/s, 213 and 257 DEGs up-regulated and down-regulated at 20 cm/s. Compared with sea urchins at 10 cm/s, there were 422 DEGs at 20 cm/s, including 211 genes up-regulated and 211 down-regulated (Fig. 4).

GO analysis was used on DEGs to further understand the physiological functions (Fig. 2B). The number of enriched DEGs between 10 and 2 cm/s showed no significant difference. Only the recombination nodule showed more enrichment between 20 and 2 cm/s. The number of DEGs was significantly higher in the recombination nodule, synaptonemal structure, and sarcoplasmic reticulum at 20 cm/s and 10 cm/s.

### Real-time quantitative PCR validation of RNA-Seq data

**Figure 5:**
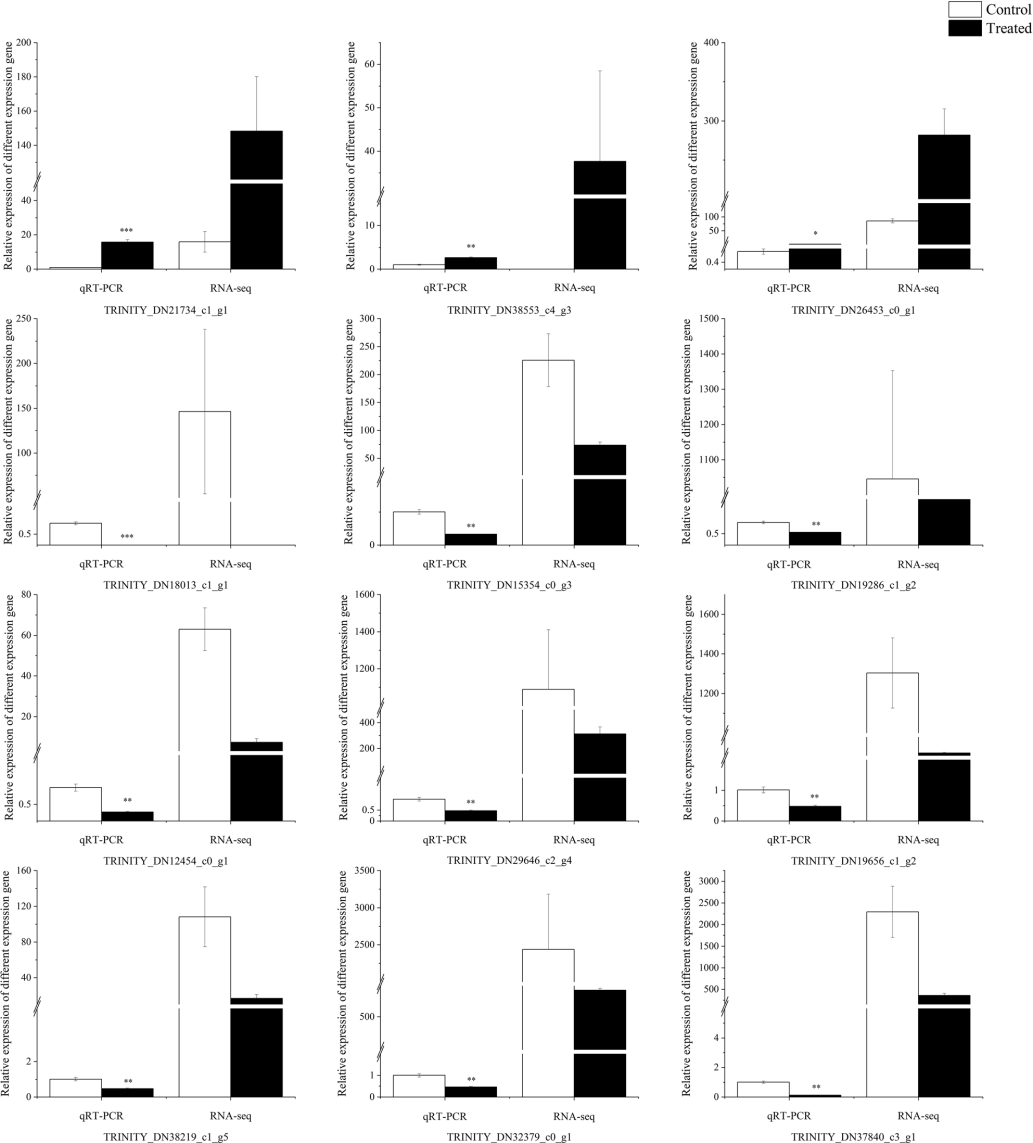
qRT-PCR verification and transcriptome sequencing of DEGs (mean ± SD). *Means *P* < 0.05, **means *P* < 0.01 and ***means *P* < 0.001.

Real-time quantitative PCR and transcriptome sequencing of differential expression multiple results are shown in Fig. 5. In the validation of 12 DEGs, qRT-PCR results showed the same change trend as the transcriptome results, indicating that the transcriptome results were reliable.

## Discussion

*Mesocentrotus* *nudus* mainly inhabit in shallow seas and intertidal zones with large changes in water flow^27^. Growth and movement commonly show great differences in *M*. *nudus* at different flow velocities^28^. However, it remains largely unclear on the molecular mechanism of the effects of flow velocities on those performances of *M*. *nudus*. The present study observed 403,298,480 clean reads and Q30 over 93.64% by transcriptome sequencing. A total of 156,177 unigenes were obtained from tube feet, which were less than that in tube feet of the sea urchin *Strongylocentrotus* *intermedius*^29^, but more than that from gonads of *M*. *nudus*^2^. This suggests that the present sequencing results are qualified and provide valuable resources for the research on the functional genes of *M*. *nudus*. The gene expression analysis found that sea urchins exposed to high flow velocity (20 cm/s) showed 470 DEGs, which are obviously less than in other environmental stressors. For example, 2125 DEGs were identified in gonads of *S.* *intermedius* at high water temperature^30^ and 29,107 DEGs were found in coelomocytes of *S.* *intermedius* in a hypoxia environment^31^. Different environmental stressors induce constant expression regulation of functional genes and consequently contribute to the cellular homeostasis^32^. The number of DEGs was less when sea urchins were exposed to a high flow velocity, indicating that high flow velocity probably causes a lower stress to sea urchins than previously being thought. The regulatory mechanisms of *M*. *nudus* may be relatively simple in response to high flow velocity. Notably, the gonad is a tissue that possess more complex metabolism than tube feet. More uniquely expressed genes were found in gonads than in tube feet of the sea urchin *Tripneustes* *gratilla*^33^. Thus, the complexity of the regulatory mechanisms is not exclusive to explain the large difference in the number of DEGs and the main reason needs to be further studied. Further, there are only 72 overlapped DEGs involved in regulation at both 10 cm/s and 20 cm/s, compared with the total number of DEGs between the groups of 10 cm/s (490 DEGs) and 20 cm/s (470 DEGs). They are important resources for further investigation.

There were 235 up-regulated and 255 down-regulated genes at 10 cm/s, and 213 up-regulated and 257 down-regulated genes at 20 cm/s, compared to at 2 cm/s. GO annotation showed that the DEGs of sea urchins were enriched in metabolic process, biological regulation and development process. Organisms make metabolic adjustments in response to different environmental stressors^30^. High flow velocity significantly affects growth and metabolism of fish. For example, it greatly increases the energy consumption of the fish *Rhynchocypris* *lagowskii*^34^ and reduces the fat content of the flounder *Paralichthys* *olivaceus*^35^. Consistently, our previous studies found that high flow velocity displays negative effects on the growth and development of sea urchins^18,28^. Here, we further found three metabolism pathways through KEGG analysis, including arachidonic acid metabolism, linoleic acid metabolism, and arginine and proline metabolism. Arachidonic acid metabolism and linoleic acid metabolism are important pathways of lipid metabolism^34^. The arginine synthesis pathway regulates the lipid metabolism through promoting oxidative decomposition of glucose and fatty acids and lipolysis of fat cells^36^. These results suggest that pathways related to lipid anabolism probably play an important role in regulating growth and development of sea urchins at high flow velocity.

Adhesion and swing of tube feet are the basis to keep balance in the foraging of sea urchins. Time for adhesion significantly extends in sea urchins at high flow velocity, because water flow reduces the swing of tube feet^28^. In the present study, 420 DEGs were found by comparing the genes expression between 10 cm/s and 20 cm/s. Transcriptome data further showed that the expression of *ankyrin-1* was significantly higher at 20 cm/s than that at 10 cm/s. The expression of *nectin-1* related to adhesion showed significant difference in sea urchins at different flow velocities, which is considered to be the key gene reducing the movement capability of sea urchins at high flow velocity^23^. *Ankyrin-1* is annotated to the sarcoplasmic reticulum, which is a master regulator of muscle contraction^37^. *Ankyrin-1* was firstly found in the preparation of erythrocyte membranes, which is an erythrocyte membrane protein that connects the underlying cytoskeleton to the plasma membrane^38^. It is consistently expressed in muscle and is annotated in other muscle activity-related GO classifications, including the Z-line^20,39^. Therefore, it is reasonable to speculate that *ankyrin-1* probably plays an important role in the movement capacity of sea urchins at different flow velocities. Higher expression of *ankyrin-1* is probably involved in mobilizing more energy of muscle to resist the stress of high flow velocity in collaboration with other genes, for example *nectin-1*. This novel finding increases our understanding on how sea urchins adapt to high flow velocity.

## Conclusion

The present study investigated the gene expressions of sea urchins at different flow velocities using transcriptomes. There were 490 and 470 DEGs at flow velocity of 10 cm/s and 20 cm/s compared to 2 cm/s, respectively. GO annotation found that a number of DEGs enriched in growth and metabolism. KEGG pathway analysis discovered three pathways related to lipid anabolism and amino acid metabolism. Thus, tube feet show different gene expression levels when sea urchins live in the habitats at high flow velocity. In addition, we found that 72 overlapped DEGs involved in regulation at both 10 cm/s and 20 cm/s. Functions of these 72 DEGs are worth further investigating to find out the molecular mechanisms of the adaptation of sea urchins exposed to high flow velocity. We highlighted a muscle-associated gene *ankyrin-1*, which is correlated with the movements of tube feet at different flow velocities. The present study provides valuable information into the molecular mechanisms of changed behaviors and growth of sea urchins exposed to high flow velocities.

## Materials and methods

### Sea urchins

*Mesocentrotus* *nudus* of 8-month age (test diameter of 21.57 ± 0.14 mm, test height of 10.12 ± 0.10 mm and body weight of 4.19 ± 0.08 g) were transported from a local aqua-farm to the Key Laboratory of Mariculture & Stock Enhancement in North China′s Sea, Ministry of Agriculture and Rural Affairs, Dalian Ocean University (121° 56′ E, 38° 87′ N). All sea urchins were then maintained in fiberglass tanks (~ 1000 m^3^) with the natural photoperiod with aeration and fed fresh kelp *Saccharina* *japonica* ad libitum. One third of seawater was renewed and feces were removed every 2 days. The water quality was measured regularly using a water quality monitoring meter (YSI, Incorporated, OH, USA). The salinity was 31.96 ± 0.05‰ and the water temperature was 19.15 ± 0.85 °C.

### Experimental design

**Figure 6:**
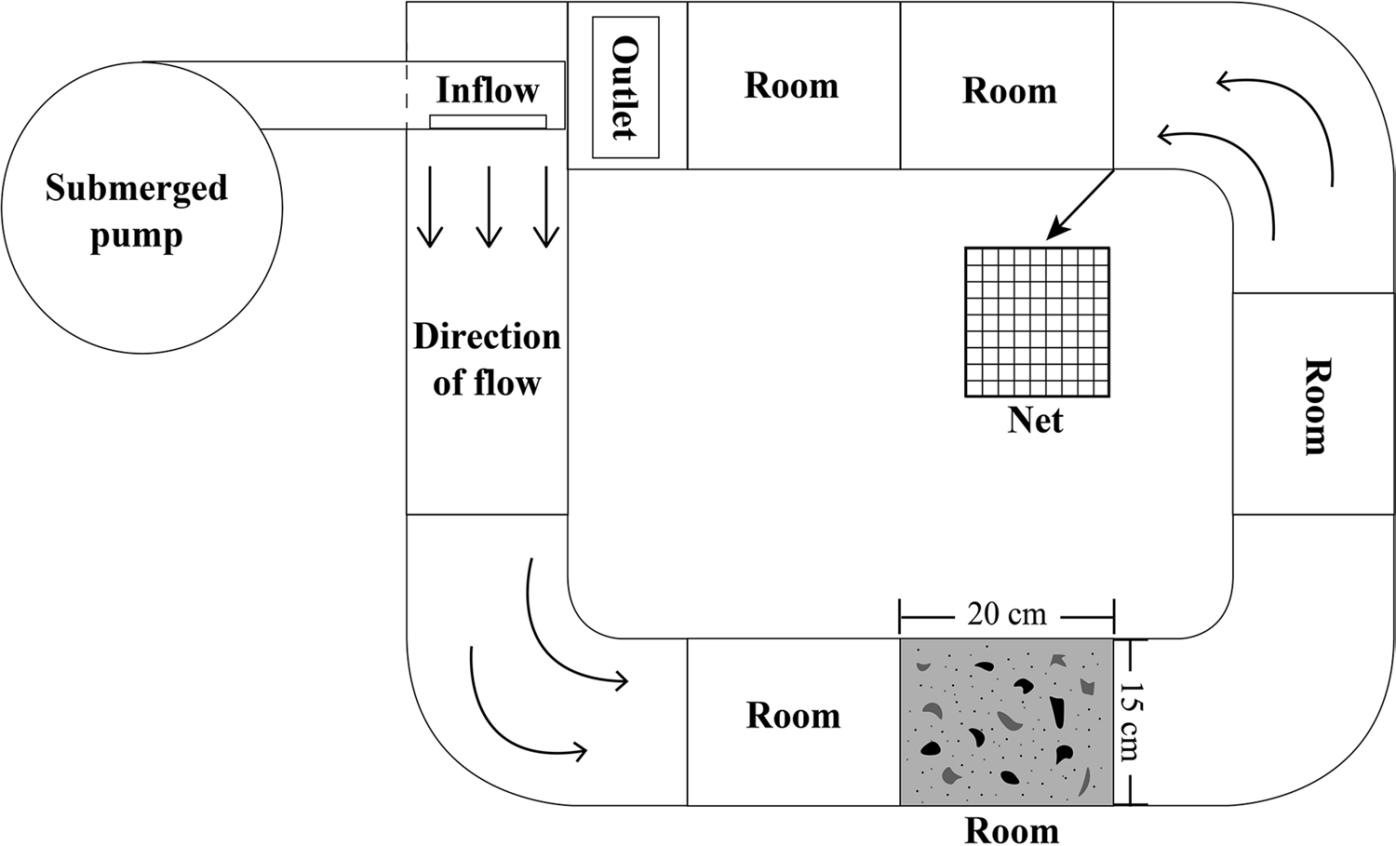
Diagram of the flow velocity experimental equipment^28^. After water flow enters from the inflow, it flows out from the outlet after entering the experimental area through the rectifier. The experimental area has 5 rooms (15 × 20 cm), separated by a net and a substrate simulation board at the bottom.

A circular runway (Fig. 6) with five rooms as experimental areas (length × width × height = 20 × 15 × 10 cm) was used in the present study^28^. The ball valve and flow meter (JDC Electronic SA Co., Switzerland) were used to control and measure flow velocity. Water circulation was supported by a water pump (200 W, 20,000 L h^−1^, Jebao, China). Sea urchins (test diameter of 21.57 ± 0.14 mm) were divided equally into three groups and cultured at three different flow velocities (2, 10, and 20 cm/s) from 7 May 2019 to 17 July 2019. There were 25 sea urchins at each flow velocity, with 5 individuals in each of the 5 rooms.

Six sea urchins were randomly selected from all sea urchins in the device. The tube feet from two sea urchins were collected in a 1.5 mL centrifuge tube and mixed as one sample to meet the test standard (12 µg) at the end of the experiment. There were three biological replicates for each experimental group (N = 3). The nine samples were immediately frozen in liquid nitrogen and stored at − 80 °C.

### RNA extraction, library construction, and RNA sequencing

Total RNAs were extracted using the conventional phenol/chloroform extraction method^40^. RNAs were diluted in a certain proportion. A micro-spectrophotometer (K5500, Beijing) and the Agilent 2100 RNA Nano 6000 Assay Kit (Agilent Technologies, CA, USA) were subsequently used to measure RNA concentration, purity, and integrity, respectively. The sample standard was OD260/280 ≥ 1.8 and OD260/230 ≥ 1.8.

The qualified samples were used for RNA library construction^41^. The mRNA was enriched by Oligo (dT) beads. The fragmented mRNA was used as the template to produce the first-strand cDNA. The first-strand cDNA was added in fragmentation buffer, dNTPs, RNaseH, and DNA Polymerase I to produce the second-strand cDNA. The products were used for the size selection by gel electrophoresis after being purified using QiaQuick PCR Kit (Qiagen, Germany). Finally, the products were amplified by PCR. The library was sequenced using Illumina Novaseq 6000 with the sequencing strategy of PE150.

The raw reads were processed through Perl scripts to ensure data quality for information analysis, including removing contaminated reads from joints (the number of nucleobase > 5 bp), removing low quality reads (Phred Quality Score ≤ 19 account for 15% of total nucleobase) and reads with N ratio greater than 5%. The data and quality of the clean reads were calculated, including Q30 and GC-content (Supplementary Table S1).

### Transcriptome assembly and gene annotation

Clean reads were assembled using Trinity software (v2.4.0)^42^ to obtain full-length transcripts (Supplementary Table S2). The length distribution and GC-content of transcripts were counted. Bowite2 (v2.2.3) was used for ratio analysis^43^ (Supplementary Table S3). Python scripts were used to analyze the uniformity of sequencing results.

The open reading frame (ORF) region for unigenes were identified using TransDecoder (v3.0.1). The annotation results of predicted ORF and transcript sequences were obtained by Trinotate (v3.0.2), including BLAST (v2.2.28), hmmScan (v.3.1b1), SignalP (v4.1), TmHMM (v2.0), and RNAmmer (v1.2). Gene Ontology (GO, http://www.geneontology.org/) analysis and euKaryotic Ortholog Groups (KOG) annotation were used to complete functional annotation^44^.

### Differential expression analysis

The method of RPKM (Reads Per Kilobase spend Mapped Reads) was used to calculate the amount of gene expressions^45^. There were three biological replicates for each flow velocity (N = 3). The differential expression analysis tool DESeq2 (v.1.6.3) was used because samples had biological repeats. The genes that |log_2_Ratio|≥ 1 and q < 0.05 were selected as DEGs (Supplementary Table S4). All DEGs were mapped to GO terms to characterize main biological functions (False discovery rate, FDR < 0.05). Kyoto Encyclopedia of Genes and Genomes (KEGG, http://www.genome.jp/kegg) pathway was used to analyze DEGs and identify the enriched metabolic pathways. GO and KEGG enrichments were analyzed using Hyper geometric test.

### mRNA library validation

Twelve highly expressed DEGs were randomly selected for validation (including three up-regulated and nine down-regulated DEGs). The *18* *S* gene was used as the reference gene and the annealing temperature was set at 60 °C^46,47^. The DEGs and *18* *S* were used for the validation of RNA-seq data by real-time PCR (Table 2). The PrimeScript™ RT reagent Kit (TaKaRa, Japan) was used for reverse transcription of the RNA of sea urchins at three different flow velocities according to the instruction and system. Fluorescence quantitative analysis was performed in Light Cycler 96 and the fluorescence quantitative kit was SYBR^®^ Premix Ex TAq™ (TaKaRa, Japan). The qRT-PCR reaction system had a total volume of 20 µL, including 2 µL of cDNA template, 10 µL of 2 × SYBR Green Master mix (TaKaRa, Japan), 0.8 µL of each primer and 6.4 µL of PCR-grade water. The running program was set as follows: 95 °C for 30 s and followed by 40 cycles. The annealing and elongation were 95 °C for 5 s and 60 °C for 32 s, respectively. PCR melting curve analysis was conducted to confirm single PCR products at the end of the reaction. The fluorescence quantitative results were calculated using the 2^−ΔΔCT^ algorithm^48^. The results of each sample were tested three times.

**Table 2:** Transcriptome-verified primers.

Genes ID	Name of genes	Primer sequence (3′–5′)	Temperature (°C)
TRINITY_DN21734_c1_g1	Deoxynucleoside triphosphate Triphosphohydrolase SAMHD1-like	F: AGGTTGTAAGAATCGCGGGCAAAG R: GAATGGACGAGAACGGGAGAAACG	60
TRINITY_DN38553_c4_g3	Ankyrin repeat domain-containing protein 50-like	F: TGGCACTACAACGAATGTGACACC R: CCCTGTTTACCTGTGCTCCTTGAC	60
TRINITY_DN26453_c0_g1	Oxysterols receptor LXR-beta isoform X1	F: CCATTGGTCTACCTCGCTTGTCAC R: TCGGTATGTTGTTCTTGCCACTGG	60
TRINITY_DN18013_c1_g1	Ankyrin repeat, PH and SEC7 domain containing protein secG-like	F: TTCCTCTCCACCAGTGCTCCTTG R: GCTTGACAATCTTGGTCGGACTCC	60
TRINITY_DN15354_c0_g3	Cilia- and flagella-associated protein 20	F: GGAGGGCATACGGCACAAACTAC R: TCGGAGTACAGTCTGTCGGAGAAG	60
TRINITY_DN19286_c1_g2	Rotatin isoform X2	F: GTCCCACATCAACCCGCATCAC R: ATCTTGGCGTTCAGCAGTTGTCTC	60
TRINITY_DN12454_c0_g1	Transmembrane protein 26	F: GGCTAAGTCCTTCGGTGGTTCTG R: CCCATCCCTGTGAGTCCCTGTAG	60
TRINITY_DN29646_c2_g4	Myb-like protein X	F: AGAGCAGCGGCAACAACAAGG R: CTGTGGACGAGGTGCTGATGATG	60
TRINITY_DN19656_c1_g2		F: AGAGCAGCGGCAACAACAAGG R: CTGTGGACGAGGTGCTGATGATG	60
TRINITY_DN38219_c1_g5	LOC105440335, ncRNA	F: AGAGCAGCGGCAACAACAAGG R: CTGTGGACGAGGTGCTGATGATG	60
TRINITY_DN32379_c0_g1	LOC100889934, mRNA	F: AGGTGATCGAAGCGGTGAATGC R: TGCTCAATCGGTCAACCAGGAATC	60
TRINITY_DN37840_c3_g1	Pol-like protein	F: ACGGCAACTGTAGTACCTGCT R: CATGTGTACCATATCAAGACCACCA	60
*18* *S*		F: GTTCGAAGGCGAGCCATCAGATAC R: CTGTCAATCCTCACTGTGTC	60

### Statistical analysis

Normal distribution and homogeneity of variance were analyzed using Kolmogorov–Smirnov test and Levene test, respectively. Gene expressions were analyzed using independent-samples *t*-test. All statistical analyses were performed by using SPSS 22.0 (Inc., Chicago, IL, USA). All data were expressed as mean values ± standard deviation (mean ± SD) and evaluated the significance at the level of *P* < 0.05.

### Ethical approval

All applicable international, national, and/or institutional guidelines for the care and use of animals were followed by the authors.

## Supplementary Information

Supplementary Tables.

## Data Availability

The raw data were submitted to the NCBI database (Accession Number: PRJNA825640).
