# Supplementary material for: Transcriptomes reveal the involved genes in the sea urchin Mesocentrotus nudus exposed to high flow velocities
Source: Sci Rep. 2022 Aug 5;12:13493. doi: 10.1038/s41598-022-17793-w (PMC9356045; doi:10.1038/s41598-022-17793-w)
Supplement: Supplementary file 1 — Supplementary Tables. [file 41598_2022_17793_MOESM1_ESM.docx]

**Supporting information**

**Article title:** Transcriptomes reveal the involved genes in the sea urchin *Mesocentrotus nudus* exposed to high flow velocity

**Author list:** Ruihuan Tian^1,+^, Dongtao Shi^1,+^, Donghong Yin^1^, Fangyuan Hu^1^, Yaqing Chang^1^, Chong Zhao^1,2,*^

^*^Email: Chong Zhao, [chongzhao@dlou.edu.cn](mailto:chongzhao@dlou.edu.cn)

| #Sample | Fv2a | Fv2b | Fv2c | Fv10a | Fv10b | Fv10c | Fv20a | Fv20b | Fv20c |
| --- | --- | --- | --- | --- | --- | --- | --- | --- | --- |
| Raw Reads Number | 49,903,064 | 52,066,824 | 48,050,592 | 46,743,776 | 48,821,320 | 46,777,512 | 46,811,970 | 47,755,126 | 45,847,236 |
| Raw Bases Number | 7,485,459,600 | 7,810,023,600 | 7,207,588,800 | 7,011,566,400 | 7,323,198,000 | 7,016,626,800 | 7,021,795,500 | 7,163,268,900 | 6,877,085,400 |
| Clean Reads Number | 47,102,826 | 47,980,862 | 45,194,736 | 43,059,178 | 45,981,134 | 44,453,294 | 44,179,722 | 44,721,852 | 40,624,876 |
| Clean Reads Rate(%) | 94.39 | 92.15 | 94.06 | 92.12 | 94.18 | 95.03 | 94.38 | 93.65 | 88.61 |
| Clean Bases Number | 7,065,423,900 | 7,197,129,300 | 6,779,210,400 | 6,458,876,700 | 6,897,170,100 | 6,667,994,100 | 6,626,958,300 | 6,708,277,800 | 6,093,731,400 |
| Low-quality Reads Number | 377,928 | 389,552 | 358,994 | 336,460 | 361,506 | 404,202 | 336,152 | 360,456 | 355,472 |
| Low-quality Reads Rate(%) | 0.76 | 0.75 | 0.75 | 0.72 | 0.74 | 0.86 | 0.72 | 0.76 | 0.78 |
| Ns Reads Number | 433,634 | 441,122 | 456,822 | 377,300 | 602,698 | 385,282 | 383,804 | 430,234 | 611,716 |
| Ns Reads Rate(%) | 0.87 | 0.85 | 0.95 | 0.81 | 1.24 | 0.83 | 0.82 | 0.91 | 1.34 |
| Adapter Polluted Reads Number | 1,988,676 | 3,255,288 | 2,040,040 | 2,970,838 | 1,875,982 | 1,534,734 | 1,912,292 | 2,242,584 | 4,255,172 |
| Adapter Polluted Reads Rate(%) | 3.98 | 6.25 | 4.25 | 6.36 | 3.84 | 3.28 | 4.08 | 4.7 | 9.28 |
| Raw Q30 Bases Rate(%) | 93.68 | 93.34 | 93.41 | 93.44 | 93.28 | 93.11 | 93.67 | 93.25 | 92.9 |
| Clean Q30 Bases Rate(%) | 94.21 | 93.9 | 93.96 | 93.94 | 93.91 | 93.64 | 94.17 | 93.81 | 93.65 |

**Supplementary Table 1.** Data filtering statistical analysis table.

| Header | Trinity | Unigene |
| --- | --- | --- |
| Count | 393,938 | 156,177 |
| Percent GC (%) | 39.01 | 38.39 |
| Total Bases | 329,618,158 | 124,272,929 |

**Supplementary Table 2.** Statistical table of assembly results.

| Read_type | Count | Percent(%) |
| --- | --- | --- |
| Total Reads | 403,298,480 | 100 |
| Aligned Reads | 332,288,136 | 82.39 |
| Pair Mapping | 271,194,264 | 67.24 |
| Singletons | 30,596,786 | 7.59 |

**Supplementary Table 3.** Statistical table of comparison results.

| Name | A_FV2 | FV10_FV2 | FV20_FV10 | FV20_FV2 |
| --- | --- | --- | --- | --- |
| Up | 339 | 235 | 211 | 213 |
| Down | 157 | 255 | 211 | 257 |
| Total | 496 | 490 | 422 | 470 |

**Supplementary Table 4.** Statistical table of number of DEGs between groups.
